# Psychosocial factors and their influence on the experience of pain: response to commentary

**DOI:** 10.1097/PR9.0000000000000607

**Published:** 2017-07-11

**Authors:** Rhiannon Edwards, Christopher Eccleston, Edmund Keogh

**Affiliations:** a Department of Psychology, University of Bath, Bath, United Kingdom; b Centre for Pain Research, University of Bath, Bath, United Kingdom

**See commentary:** Tracy LM. Psychosocial factors and their influence on the experience of pain. PAIN Reports 2017:e602.

We thank the author for their interest and comments regarding our recent study. We agree that further work is required to tease out, and better understand, the relevant psychosocial mechanisms. You highlight some very interesting ideas, particularly around fear of pain and catastrophizing. Constructs such as empathy and competition–cooperation, and the impact on interpersonal interactions are potentially relevant also. We view this as the starting point for an interesting line of research, and wish to build on these ideas to aid our understanding of the role that social and contextual factors have on pain.

